# The Potential of CRISPR/Cas9 Gene Editing as a Treatment Strategy for Alzheimer’s Disease

**DOI:** 10.4172/2161-0460.1000439

**Published:** 2018-05-31

**Authors:** Troy T Rohn, Nayoung Kim, Noail F Isho, Jacob M Mack

**Affiliations:** Department of Biological Sciences, Science Building, Room 228, Boise State University, Boise, Idaho, USA

**Keywords:** Alzheimer’s disease, CRISPR/Cas9, Gene editing, Treatment, Huntington’s disease, iPSC neurons

## Abstract

Despite a wealth of knowledge gained in the past three decades concerning the molecular underpinnings of Alzheimer’s disease (AD), progress towards obtaining effective, disease modifying therapies has proven to be challenging. In this manner, numerous clinical trials targeting the production, aggregation, and toxicity of beta-amyloid, have failed to meet efficacy standards. This puts into question the beta-amyloid hypothesis and suggests that additional treatment strategies should be explored. The recent emergence of CRISPR/Cas9 gene editing as a relatively straightforward, inexpensive, and precise system has led to an increased interest of applying this technique in AD. CRISPR/Cas9 gene editing can be used as a direct treatment approach or to help establish better animal models that more faithfully mimic human neurodegenerative diseases. In this manner, this technique has already shown promise in other neurological disorders, such as Huntington’s disease. The purpose of this review is to examine the potential utility of CRISPR/Cas9 as a treatment option for AD by targeting specific genes including those that cause early-onset AD, as well as those that are significant risk factors for late-onset AD such as the apolipoprotein E4 (APOE4) gene.

## An Introduction to Alzheimer’s Disease (AD)

Alzheimer’s Disease (AD) is a progressive and fatal neurodegenerative disorder that primarily affects older adults and is the most common cause of dementia [1]. Currently it afflicts 5.5 million Americans and that number is expected to triple by 2050. At the present time, it is the third leading cause of death behind heart disease and cancer, with an estimated 700,000 Americans ages>65 years will have AD when they die [2]. In addition, the cost of the disease is substantial with $259 billion health care dollars going to manage the disease currently, and by the middle of the century costs are predicted to soar over $1.2 trillion, which will completely bankrupt the healthcare system in the USA [3]. Worldwide, 47 million people live with dementia and that number is projected to increase to more than 131 million by 2050 with an estimated worldwide cost of US $818 billion [4].

Clinically, patients with AD most commonly present with insidiously progressive memory loss, to which other spheres of cognition are impaired over several years. In addition to memory loss, patients may also experience language difficulties (e.g. anomic aphasia) and loss of executive skills, symptoms that epitomize the all-encompassing term dementia. In essence, AD interferes with memory, thinking, and behavior severely enough to affect a person’s work, hobbies, and social life. AD is inexorably progressive and fatal within 5 to 10 years [5].

The classic neuropathological footprints of AD are characterized by extracellular senile plaques composed of beta-amyloid and intracellular lesions of truncated and hyperphosphorylated tau leading to neurofibrillary tangles (NFTs) [5]. Importantly, it is the loss of synapses that is best correlated with the degree of dementia [6–8]. Imaging studies in autosomal dominant AD brains have documented early accumulation of beta-amyloid on PET scans as early as 15–20 years before symptoms became evident [9,10], suggesting there is, potentially, a large therapeutic window to intercede. These studies have reinforced the rationale behind the beta-amyloid hypothesis, which suggests that beta-amyloid in the form of toxic oligomers is thought to be the key initiating species leading to all downstream events culminating in synapse loss, neurodegeneration and finally, dementia [11–14].

The advent of the beta-amyloid hypothesis provided the impetus for the development and testing of the first known disease-modifying therapeutics that collectively had an action of either preventing beta-amyloid from forming or led to enhanced clearance out of the brain [15]. Unfortunately, these clinical trials have failed resoundingly with more than 400 failed clinical trials since the last Alzheimer’s drug-which only temporarily treats the symptoms of the disease–was approved more than a decade ago. A recent analysis of clinical trials involving Alzheimer’s disease indicated a very high attrition rate with an overall success rate during the 2002 and 2012 period of 0.4% (99.5% failure) [16]. Although there are many plausible reasons for these clinical trial failures, one prevailing view is that patients were too far advanced in the disease process for anti-beta-amyloid drugs to have an impact on cognition. Due to the numerous failures reported for the current disease-modifying approaches, now may be time to earnestly focus on other potential treatment paradigms including gene editing systems. Currently, there are three commonly used gene editing tools available to researchers including zinc finger nucleases (ZFNs), transcription activator-like effectors nucleases (TALENs), and the clustered regularly interspaced short palindromic repeats (CRISPR), CRISPR-associated nuclease 9 (CRISPR/Cas9). All three of these gene editing tools have their advantages and disadvantages [17]; however, this review will focus on the utility of the CRISPR/Cas9 because it is faster, cheaper, more accurate and efficient than other existing genome editing methods. Moreover, the success of this technique has recently been demonstrated in Huntington’s disease as will be described below.

## The Basics of CRISPR/Cas9 and its Application in a Representative Neurodegenerative Disease

**Figure 1:**
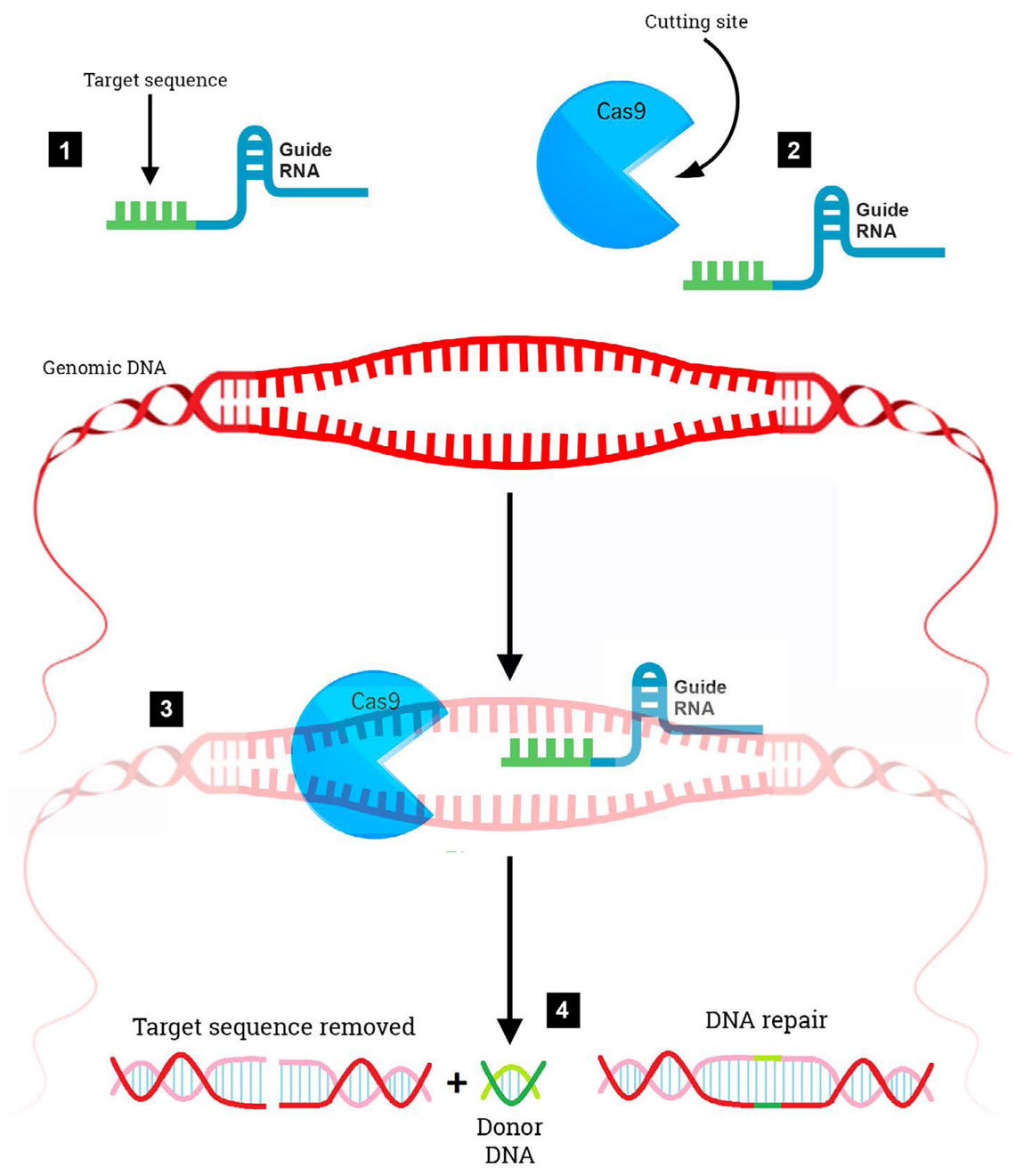
Editing a gene using the CRISPR/Cas9 Technique. A schematic cartoon illustrating that shows the potential of CRISPR/Cas9 to treat and/or cure potential human disease. The first step is to replace the mutation with the wild-type sequence, which occurs in four separate steps: Step 1 is creating the guide RNA that matches the piece of DNA in the genome that contains the mutation. Step 2 is combining this sequence along with the enzyme Cas9, which is a DNAase capable of cleaving both strands of the DNA double helix. Step 3 is combining the guide RNA and Cas9 with the appropriate cell containing the genomic DNA. The Cas9 will search the genomic DNA for the correct sequence using the guide RNA and once found, will selectively clip out the targeted sequence. Not shown in Step 3 are short DNA sequences known as protospacer adjacent motifs (PAMs) that serve as tags and sit adjacent to the target DNA sequence. In Step 4, once the DNA is cut, the cell’s natural repair mechanisms fix the break by filling in the gap with a sequence of nucleotides of choice (i.e., the wild-type sequence), thereby correcting a mutation.

The RNA-guided clustered regularly interspaced short palindromic repeats/CRISPR associated nuclease 9 (CRISPR/Cas9) system is a revolutionary genome editing tool derived from the bacterial Type II CRISPR adaptive immune system [18,19]. The CRISPR/Cas9 system was found to target and cut specific DNA sequences using only a nuclease and RNAs to target specific DNA sequences (Figure 1). Its primary function in bacteria is the endonucleolytic destruction of invading plasmid or phage DNA [20]. Cas9 recognizes a very short conserved sequence (a few nucleotides in length) adjacent to the guide spacer sequence known as “Protospacer Adjacent Motif” (PAM). These sequences have homology to viral genes and all share common sequences at one end. Subsequently, it was found that PAM is required for target recognition [21]. Once directed to the DNA target site, Cas9 generates a double strand break, resulting in gene knockout effects of template-dependent gene replacement (Figure 1). Key to this system is a piece of RNA called the guide RNA. This consists of a small pre-designed RNA sequence (about 20 bases long) located within a longer RNA scaffold. The scaffold part binds to DNA and the predesigned sequence then guides Cas9 to the correct part of the genome (Figure 1) [22]. This implies, at least in theory that the guide RNA will only bind to the target sequence and no other regions of the genome. At this stage, DNA repair mechanisms recognize the cleaved DNA and repair by cellular DNA repair mechanisms, either via the non-homologous end joining DNA repair pathway or the homology directed repair pathway [23]. The CRISPR/Cas9 system has advantages over other gene editing tools (zinc finger nucleases or transcription activator like-effector nucleases) including ease of use, low cost, and its ability to generate gene knockouts, knockins, or smaller mutations. The potential applications of CRISPR/Cas9 are far reaching including being able to precisely and efficiently target, edit, modify, regulate, and mark genomic loci of a wide array of cells and organisms. Its application in genome-wide studies will enable large-scale screening for drug targets and other phenotypes and will facilitate the generation of engineered animal models that will benefit the understanding of human diseases. The ultimate goal of this system would be to correct mutations at precise locations in the human genome in order to treat genetic causes of disease including certain neurodegenerative disorders.

## Potential Application of CRISPR/Cas9 in the Treatment of Neurological Disorders

**Figure 2:**
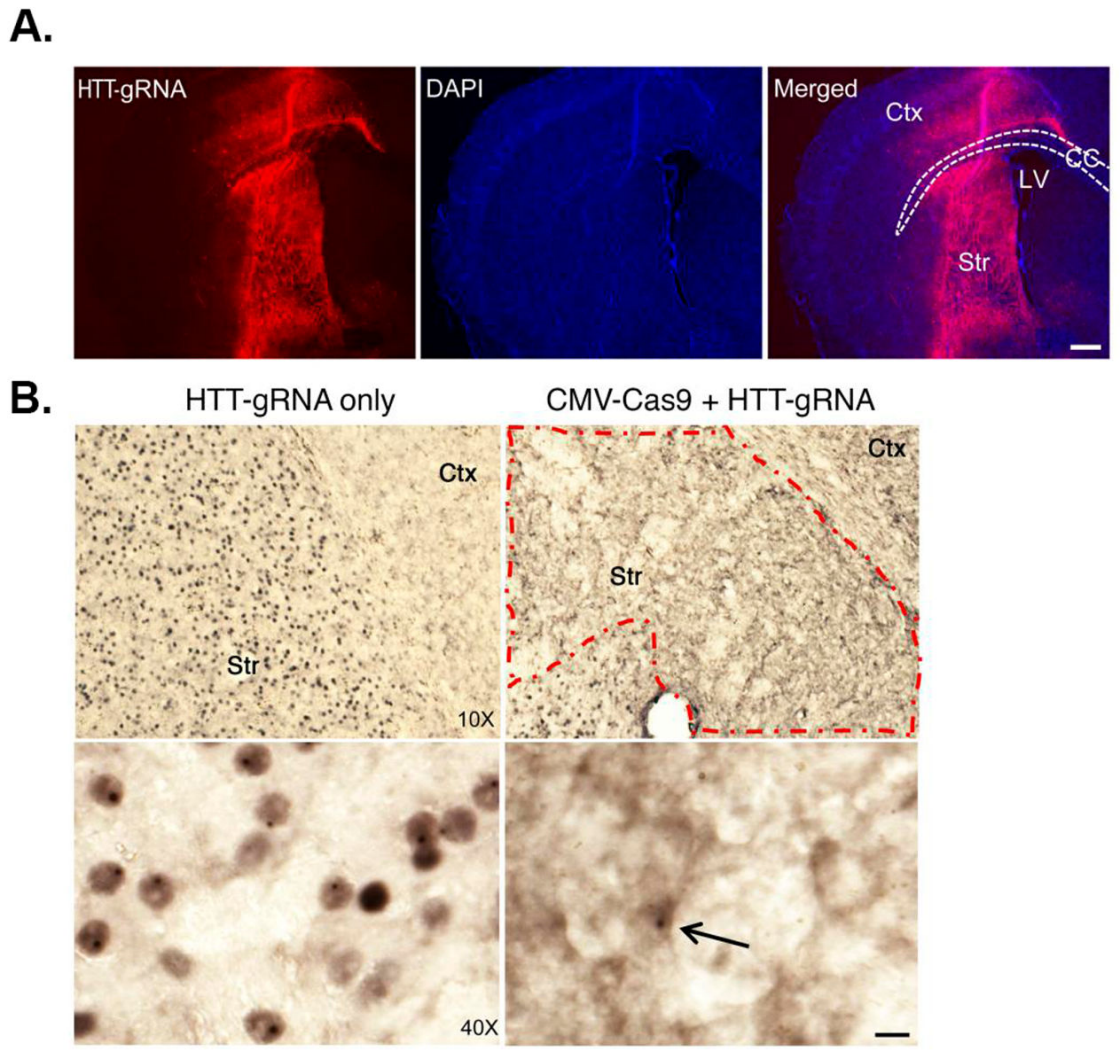
CRISPR/Cas9 gene editing reduces pathology in a mouse model of Huntington’s disease. (A): Immunofluorescence showing the transduction of AAV-HTT-gRNA in the striatum and part of the cortex. Ctx, cortex; Str, striatum; CC, corpus callosum; LV, lateral ventricle. Scale bar: 100 μm. (B): Low- and high-magnification images show the reduction of nuclear HTT and HTT aggregates in the AAV-HTT-gRNA/AAV-CMV-Cas9–injected area in 9-month-old homozygous HD140Q-KI mice compared with the contralateral striatum injected with AAV-HTT-gRNA only. Arrow indicates a remaining cell with nuclear HTT inclusion. Scale bar: 10 μm. The red dashed outline indicates the injected region where mHTT aggregates are markedly reduced.

The CRISPR/Cas9 technique has shown promise in certain neurological disorders, most notably Huntington disease (HD). HD is caused by the expansion of CAG repeats in exon 1 of the HTT gene, which encodes for mutant huntingtin (mHTT), a large protein consisting of large polyglutamine repeats in the N-terminal domain of mHTT that most likely exhibits a toxic-gain of function [24,25]. One potential strategy to treat HD could be using CRISPR/Cas9 to selectively suppress the expression of mHTT. The rationale for this approach comes from a previous study demonstrating that the application of RNA interference improved motor and neuropathological abnormalities in a HD mouse model [26]. Since this study, the CRISPR/Cas9 gene editing system has been successfully applied to HD [27–29]. Recently, CRISPR/Cas9 was used to selectively suppress the entire HTT and mHTT gene in an *in vivo* mouse model of HD (non-allele specific approach) [30]. This was achieved due to previous studies that showed depletion of normal HTT in adult mouse brains does not affect animal survival, growth, or neuronal viability [31]. These data suggested that removal of N-terminal HTT containing the polyQ domain, regardless of its allele, could be a potential therapeutic strategy to treat HD. The main challenge of using CRISPR/Cas9 in this mouse model of HD is targeting the gene-editing system to the appropriate area of the brain. This was achieved by using adeno-associated virus (AAV) and injecting viral vectors carrying the CRISPR/Cas9 into the striatum region of the brains of HD mice at 9 months of age. Yang et al. demonstrated that most of the striatum in the cortex were transduced by AAV-HHT-gRNA after injection of AAV-HHT-gRNA and AAV-CMV-Cas9 into one side of the striatum in 9-month-old homozygous HD140Q-KI mice (Figure 2) [30]. This led to a dramatic decrease in aggregated mHTT in the striatum three weeks later (Figure 2). Importantly, the authors also demonstrated improved motor performance including balance and grip in treated mice, although they never recovered as well as control mice [30]. Finally, the authors noted few other potential off-target gene effects and the gene editing appeared to be specific to Htt/mHTT. This was an important finding, as a major concern about CRISPR/Cas9 gene editing is off-target effects. Recently, an extension of this study using the CRISPR/Cas9 gene editing system examined a new variant of the gene-editing system that appears to be safer and more specific by using a nickase version of Cas9 [32]. The apparent advantage of this version of Cas9 is it cuts just one DNA strand instead of two, which increases the precision with which Cas9 can edit sequences of DNA. The authors demonstrated that the CAG repeat can be precisely excised from the HTT gene with the use of this Cas9 nickase strategy. This resulted in abrogation in huntingtin synthesis in HD patient-derived fibroblasts [32].

Overall, these findings support the utility of using CRISPR/Cas9 to correct mutant protein expression in specific brain regions and opens up the possibility of a new treatment strategy not only for HD but other neurodegenerative diseases that stem from mutant genes, including AD.

## Potential Therapeutic Applications of CRISPR/Cas9 in Treating Alzheimer’s Disease

### Evidence in early-onset AD models

**Figure 3:**
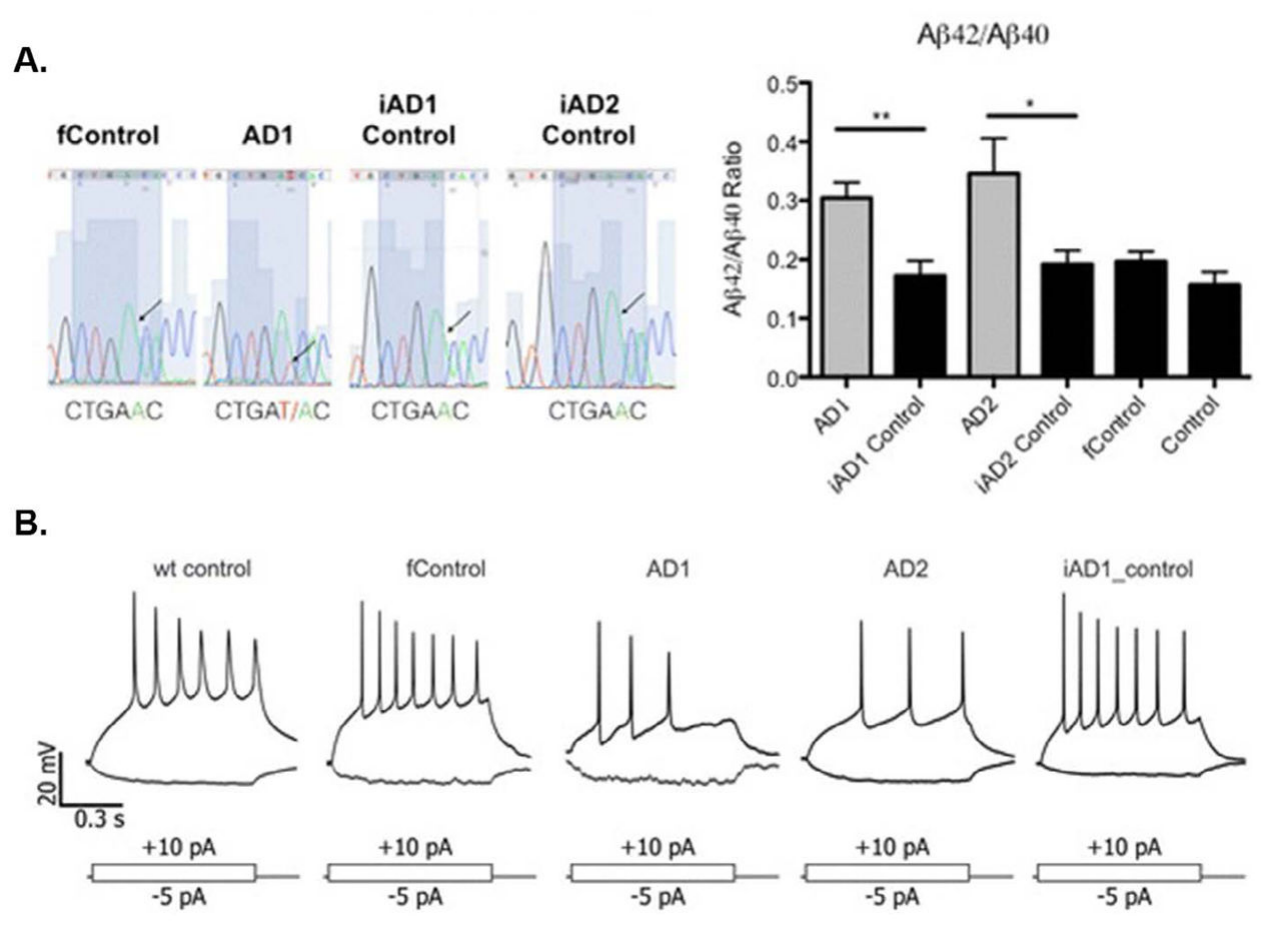
CRISPR/Cas9 corrects Alzheimer’s disease mutation in basal forebrain cholinergic neurons derived from iPSCs. (A): Left panel shows Sanger sequencing results from iPSC lines, showing corrections in the N141I mutation. Right panel depicts normalization of Aβ 42/40 ratio in CRISPR/Cas9 corrected BFCNs. (B): CRISPR/Cas9 correction of PSEN2 mutation abolishes electrophysiological deficits, restoring both the maximal number of spikes and spike height to the levels recorded in controls.

Because the majority of AD cases are sporadic with the trigger unknown, the use of CRISPR/Cas9 would not seem a viable treatment approach. This is supported by the fact that a very small percentage of cases (<1%) are caused by known mutations in the APP protein or genes products involved in processing APP to form beta-amyloid. What is certain is that, although these mutations make up a small percentage of known AD cases, they all lead to enhanced production of the beta-amyloid peptide [33]. Other known mutations that lead to early-onset AD include those to presenilin 1 (PSEN1) and presenilin 2 (PSEN2) [34,35]. The net effect of mutations to these two genes is enhanced production of beta-amyloid (1-42) perhaps by shifting the cleavage site in APP [36]. The majority of these cases manifest before the age of 60 and are, therefore are classified as early-onset AD. Clearly, the potential for CRISPR/cas9 in potentially correcting these autosomal-dominant mutations is real and could be pursued. This is supported by recent studies that have analyzed the potential of correcting similar kinds of mutations using this gene editing system. For example, CRISPR/Cas9 was used to correct a presenilin (PSEN2) autosomal dominant mutation in iPSC-derived neurons. In this study, the authors generated basal forebrain cholinergic iPSC neurons from an individual carrying the PSEN2N141I mutation [37]. As shown in Figure 3 [37], CRISPR/Cas9 corrected the N141I mutation demonstrated by Sanger sequencing that led to a normalization of the Aβ 42/40 ratio (Figure 3A). Functionally, the CRISPR/Cas9 correction of the PSEN2 mutation reversed electrophysiological deficits (Figure 3B). This study was supported by previous studies that have also used CRISPR/Cas9 to correct familial AD mutations in the PSEN gene in patient-derived iPSCs [38,39].

Recently, a new study reported on how CRISPR/Cas9 was used to knock out the Swedish APP mutation in patient-derived fibroblasts leading to a 60% reduction in secreted beta-amyloid [40]. The only known mutation immediately adjacent to the β-secretase site in APP is the Swedish mutation, which is actually a double mutation that results in a substitution of the two amino acids, lysine and methionine to asparagine and leucine [41]. The authors also sought to disrupt this mutation in vivo using Tg2576 mice, which carry multiple copies of the APP Swedish mutation. To accomplish this, they injected DNA encoding both Cas9 and guide RNAs in AAV vectors into the hippocampus of transgenic mice. Although it remains to be seen if such manipulation will decrease the pathology and behavior deficits associated with Tg2576 mice, the authors were able to show some disruption of the APP Swedish gene, mostly in the form of single base pair insertions. It’s noteworthy, however, that the direct injection of CRISPR/Cas9 system into hippocampus led to a very low percentage (on average 2%) of transgenes disrupted within the injected area [40]. This could be due to the fact that the Tg2576 mice harbor ~100 copies of the transgene per neuron, indicating that the level of CRISPR/Cas9 was insufficient to correct the Swedish mutation in an overexpression system. Overall, although tantalizing in its promise, a more systematic analysis of the targeted cells in the hippocampus needs to be undertaken to understand the low editing efficiency in vivo.

### Evidence and potential in sporadic AD models

These studies certainly set the table for future studies examining whether CRISPR/Cas9 gene editing can be used in patients with early onset AD, but what about sporadic AD, which by far, represents the vast majority of cases in the USA? In a preprint article that has not been peer-reviewed, Sun et al. attempted to edit endogenous APP at the extreme C-terminus to attenuate β-cleavage and beta-amyloid production [42]. In essence, they utilized CRISPR/Cas9 editing to target elimination of the C-terminal region of APP. The rationale for this approach is, as the authors have previously demonstrated, clipping off the C-terminal region of APP prevents a subsequent interaction with BACE-1 within endosomes, which is the first important cleavage event in generating beta-amyloid [43]. Their current findings in cell lines, cultured neurons, human iPSC-neurons and mouse brains all demonstrated the strategy works by limiting the physical association of APP with BACE-1 and attenuating beta-amyloid production [42].

The other major risk factor for developing late-onset AD is harboring the apolipoprotein E4 (APOE4) allele [44]. Human apoE is polymorphic with three major isoforms, apoE2, apoE3, and apoE4, all of which differ by single amino acid substitutions involving cysteine-arginine replacements at positions 112 and 158 [45]. The E2 allele is the rarest form of APOE and carrying even one copy appears to reduce the risk of developing Alzheimer’s by up to 40%. The APOE3 is the most common allele and doesn’t seem to influence risk, while the APOE4 present in approximately 10–15% of people, increasing the risk for AD and lowering the age of onset [46]. Having one copy of E4 (E3/E4) can increase your risk 2 to 3 times, while two copies of E4 (E4/E4) can increase the risk by 10–15 times [44]. It is noteworthy that 65–80% of all AD patients have at least one APOE4 allele [46,47]. Finally, although many of the adverse effects of APOE4 appear to be associated with beta-amyloid [48], a recent study supports that apoE4 may promote pathology such as tau phosphorylation in human iPSC-derived neurons, independent of beta-amyloid [49]. The authors also showed that converting apoE4 to apoE3 by gene editing (utilizing zinc-finger nuclease-mediated gene editing not CRISPR/Cas9) prevented the pathology associated with apoE4 in their model system [49]. Therefore, one potential use of the CRISPR/Cas9 system could be to convert APOE4 to APOE2 or E3. In this regard structural and functional changes from apoE4 to apoE3 or apoE2 mediated through CRISPR/Cas9 may be a viable approach to treat AD patients carrying APOE4. Interestingly, although amino acid residues 112 and 158 (cysteine to arginine substitutions) determine the different isoforms of APOE, evidence suggests a major structural characteristic of apoE4 that distinguishes it from apoE2 and apoE3 is a domain interaction mediated by a salt bridge between Arg-61 and Glu-255 [50]. Therefore, the use of CRISPR/Cas9 to alter any one of these two amino acids may also be as effective in neutralizing the risk associated with harboring the APOE4 allele.

## Conclusion

CRISPR/Cas9 can target virtually any gene in a sequence-dependent manner, and its targeting efficiency is higher than other gene targeting approaches. Thus, this system could be applied to any number of autosomal-dominant mutations that give rise to early-onset AD, or for those genetic risk factors that enhance the dementia risk associated with late-onset AD, in particular the APOE4 allele. However, challenges including off-target effects and targeting CRISPR/Cas9 to specific cell types in the CNS may prove difficult as the best option at this time is through viral vectors such as adeno-associated virus (AAV) [51]. Whether the hope of CRISPR/Cas9 as a therapeutic tool to treat AD will be realized remains to be seen, much more research is necessary to improve the technique and to demonstrate proven efficacy in animal models of AD.
